# Effect of veterinary feed directive rule changes on tetracycline-resistant and erythromycin-resistant bacteria (*Salmonella*, *Escherichia*, *and Campylobacter*) in retail meats in the United States

**DOI:** 10.1371/journal.pone.0289208

**Published:** 2023-08-03

**Authors:** Shamim Sarkar, Chika Okafor

**Affiliations:** Department of Biomedical and Diagnostic Sciences, College of Veterinary Medicine, University of Tennessee, Knoxville, TN, United States of America; University of Illinois Urbana-Champaign College of Veterinary Medicine, UNITED STATES

## Abstract

**Background:**

Antimicrobial-resistant bacteria are a growing public health threat. In 2017 the U.S. Food and Drug Administration implemented Veterinary Feed Directive (VFD) rules changes to limit medically important antimicrobial use in food-producing animals, combating antimicrobial-resistant bacteria. The effect of the VFD rule changes on the occurrence of bacteria resistant to medically-important antimicrobials in retail meats is yet to be investigated in the U.S. This study investigates whether the VFD rule changes affected the occurrence of tetracycline-resistant and erythromycin-resistant bacteria (*Salmonella*, *Escherichia*, *and Campylobacter*) in retail meats in the U.S.

**Methods:**

Multivariable mixed effect logistic regression models were used to analyze 2002–2019 retail meats surveillance data from the National Antimicrobial Resistance Monitoring System (NARMS) in the U.S. Variables included VFD rule changes, meat type, quarter of year, and raising claims. A potential association between these variables and the occurrence of tetracycline-resistant and erythromycin-resistant bacteria (*Salmonella*, *Escherichia*, *and Campylobacter*) in retail meats was estimated.

**Results:**

Analysis included data regarding tetracycline-resistant *Salmonella* (n = 8,501), *Escherichia* (n = 20, 283), *Campylobacter* (n = 9,682), and erythromycin-resistant *Campylobacter* (n = 10,446) in retail meats. The odds of detecting tetracycline-resistant *Escherichia* (OR = 0.60), *Campylobacter* (OR = 0.89), and erythromycin-resistant *Campylobacter* (OR = 0.43) in chicken breast significantly decreased after the VFD rule changes, compared to the pre-VFD rule change period. The odds of detecting tetracycline-resistant *Salmonella* (0.66), *Escherichia* (OR = 0.56), and *Campylobacter* (OR = 0.33) in ground turkey also significantly decreased. However, the odds of detecting tetracycline-resistant *Salmonella* (OR = 1.49) in chicken breast and erythromycin-resistant *Campylobacter* (OR = 4.63) in ground turkey significantly increased. There was no significant change in the odds of detecting tetracycline-resistant *Salmonella* and *Escherichia* in ground beef or pork chops.

**Conclusions:**

The implementation of VFD rule changes had a beneficial effect by reducing the occurrence of tetracycline-resistant and erythromycin-resistant bacteria in chicken and ground turkey. Ongoing surveillance of antimicrobial resistance and antimicrobial use could complement the implementation of stewardship such as VFD rule in food-producing animals in the U.S.

## Introduction

Animal-sourced protein consumption has increased globally over the last decade [1]. The animal-sourced protein consumed in the United States (U.S.) mostly comes from the industrial food-animal production system [2], which is characterized by large-scale, densely stocked conditions [3], and the widespread usage of antimicrobials to treat sick animals and control disease [4,5]. Before the implementation of the Veterinary Feed Directive (VFD) rule changes in the U.S., antimicrobials were commonly used in food-producing animals as preventive measures. However, since the VFD rule changes were implemented, antimicrobials can no longer be used in a prophylactic manner [4].

Antimicrobial resistance is a global health concern [6,7]. Multiple factors are associated with the emergence of antimicrobial resistance bacteria [8]. Overuse and misuse of antimicrobials are important factors associated with resistance to antimicrobial drugs [9,10]. The use of antimicrobials in food-producing animals in the U.S. varies across different classes of antimicrobials. In the U.S., specifically in 2020, tetracycline was the most frequently used antimicrobial, representing 66% (3,948,745 kg) of total use and 7% (433,394 kilograms) of macrolides in food-producing animals [11]. Antimicrobial resistance has emerged due to the selective pressure exerted by antimicrobial use in food-animal production [12–14]. The improper use of antimicrobial drugs in food-producing animal farming has increased antimicrobial resistance bacteria in animal food products [15,16]. The dose, route, duration, and class of antimicrobials (selective pressure) are important variables that may affect the development of antimicrobial resistance [17,18]. Also, antibiotic usage, whether for therapeutic, prophylactic, or growth promotion purposes, all contribute to the selection pressure leading to antibiotic-resistant development [19,20]. Reducing the misuse of antibiotics, whether for therapeutic, growth promotion, or prophylactic purposes, may strengthen the prevention and control of the development of antimicrobial resistance [18]. Biologically, reducing antimicrobial use can decrease the selection pressure for developing resistance [21]. For example, the resistance level in hospital-acquired pathogens may change rapidly within weeks or months after reforms in antimicrobial use [22]. Multifaceted interventions are needed to reduce the improper use of antimicrobials [23] and promote the judicious use of medically important antimicrobials in food-producing animals to control the development of antimicrobial resistance. Resistance to tetracycline and erythromycin were selected for the current analysis because these are medically important antibiotics [24] approved for use in food-producing animals in the U.S. [25].

The VFD rule changes are an important strategy to ensure the judicious use of medically important antimicrobials in food-producing animals in the U.S. On October 1, 2015, the U.S. Food and Drug Administration (FDA) changed the rules for the VFD. The rules were fully enforced on January 1, 2017, following the FDA’s Guidance for Industry # 2013 [4]. This VFD rule change highlighted the FDA’s concerns about developing antimicrobial resistance (AMR) pathogenic bacteria in humans and animals when medically important antimicrobial drugs are imprudently used in food-producing animal production. The changes made to the VFD rules limit the use of medically important antimicrobial drugs given to animals via feed and water, only allowing usage for treating illness. The VFD has impacted a number of medically important antimicrobial classes, such as tetracyclines, macrolides, and penicillins. However, it is important to note that these antimicrobials can still usage for therapeutic purposes through the administration via drinking water under the supervision of a licensed veterinarian [26,27]. The VFD rule changes have reduced the presence of violative sulfonamide and penicillin residues in the tissue of food animals at U.S. slaughterhouses, compared to before the VFD rule changes [28]. The effect of the VFD rule changes on the occurrence of bacteria resistant to medically important antimicrobials in retail meats is yet to be investigated in the U.S.

The National Antimicrobial Resistance Monitoring System (NARMS) was set up in 1996 as a joint initiative involving state and local public health departments, universities, the FDA, the Center for Disease Control and Prevention (CDC), and the U.S. Department of Agriculture’s (USDA) [29]. This national surveillance system has routinely collected data on the antimicrobial susceptibility of enteric bacteria from meats sold in stores since 2002 [29]. This program aims to monitor changes in the antimicrobial susceptibility of enteric bacteria in the US., with a specific component of the surveillance being devoted to retail meats.

Meat and meat products can be an important source of exposure to antimicrobial-resistant bacteria in humans [30–33]. Numerous studies have indicated the existence of antimicrobial-resistant bacteria found in meats sold at retail stores globally [31,34–39]. Antimicrobial-resistant bacteria originating from meats can be transmitted to humans through direct contact with meats or the consumption of meat products [40,41]. In cases where humans contract antimicrobial-resistant bacteria, the resulting infection may be challenging to manage or lead to unfavorable clinical consequences [42,43]. Foodborne illness associated with enteric bacteria is a significant public health issue in the U.S. Approximately 1 in 6 Americans suffer annually from a foodborne illness, resulting in almost 48 million cases, 128,000 hospitalizations, and 3000 deaths in the U.S. [44]. Three food-associated enteric bacteria, namely *Salmonella*, *Escherichia*, *and Campylobacter*, are responsible for approximately 60% of these illnesses and hospitalizations in the U.S. [44,45]. These bacteria also infect food-producing animals, making them a suitable focus for the current study.

Several studies have been conducted on the prevalence of antimicrobial-resistant bacteria in retail meats in the U.S. Most of these studies aimed to characterize and assess the profiles of antimicrobial-resistant bacteria isolates in retail meats, using cross-sectional surveys conducted in the U.S. between 1998 and 2018 [32,46–48]. A recent study utilized a subset of NARMS data covering the period from 2008 to 2017 and compared *Salmonella* prevalence and antibiotic susceptibility profiles in retail poultry meats with and without antibiotic-related claims [49]. Another study explored the associations between meat production methods and AMR and bacterial contamination of retail meats, using NARMS data covering 2012 to 2017 [50]. However, recent studies utilizing NARMS data did not evaluate the potential impact of VFD rule changes on the risk of antimicrobial-resistant bacteria in retail meats in the U.S. Therefore, our study’s objective was to investigate the effect of the 2017 VFD rule changes on the occurrence of tetracycline and erythromycin-resistant bacteria (*Salmonella*, *Escherichia*, *and Campylobacter*) in the U.S. using the NARMS dataset. The findings of this study will provide evidence of the magnitude of the impact that VFD rule changes have had on the risk of AMR bacteria isolates in retail meats in the U.S.

## Materials and methods

### Data source and retrieval

On March 1, 2023, the retail meats surveillance dataset from 2002 to 2019 was downloaded from the NARMS, which is publicly available data [51]. Under this surveillance system, chicken breast, ground beef, ground turkey, and pork chops are collected from grocery stores and tested for bacterial contamination and their resistance to antimicrobial drugs [51]. Subsequently, the obtained data was transferred from Microsoft Excel to STATA 17.1 software (Stata Corporation, College Station, TX, USA) for data validation and analysis.

### Data preparation and variables

The primary variable of interest utilized in selecting the final dataset for analysis was the presence of the minimum inhibitory concentration (MIC) values of tetracycline and erythromycin. The other selected variables for analysis in each dataset included month, year, state, meat type, raising claims (regarding how the animals were raised and whether they were given antibiotics), and type genera of bacteria found (*Salmonella*, *Escherichia*, *and Campylobacter*). The bacteria categories analyzed in this study include *Salmonella*, *Escherichia*, and *Campylobacter*, which encompass the aggregation of their respective serotypes. The “raising claims” variable has numerous text-form responses, which was further categorized into three groups (conventional, without any antibiotics, and not specified). Responses that included words such as “organic”, or “no antibiotic” or “antibiotic-free” (without any antibiotics) were classified together as “without any antibiotics” categories [52]. Likewise, responses without specific antibiotic-free claims such as “all-natural”, “use antibiotic responsibly”, “grass-fed” were grouped together as “conventional”. Similarly, responses containing the words “none” or “not specified” were grouped together as "not specified". After analyzing the data, we found that there was no difference between the conventional and not specified categories. As a result, we merged the not specified category with the conventional category. The final raising claims were either ‘without any antibiotic’ or ‘conventional’. The “year” variable was treated as an individual year variable. Also, we collapsed “year” variable into a categorical variable called “years of sampling”. This categorization was based on three years: ‘2002–2004’, ‘2005–2007’, ‘2008–2010’, ‘2011–2013’, ‘2014–2016’, and ‘2017–2019’. Additionally, we also created a dichotomous variable called “VFD rule change,” which collapse the “year” variable into two categories: “before VFD rule change (2002–2016)” and “after VFD rule change (2017–2019).” The “VFD rule changes” is a two-level categorical variable, with ‘after VFD rule changes’ meaning the retail meats were sampled and tested between 2017 and 2019, and ‘before VFD rule changes’ meaning the retail meats were sampled and tested between 2002 and 2016.

The "month" variable was categorized into four quarters: Quarter 1 (January, February, and March), Quarter 2 (April, May, and June), Quarter 3 (July, August, and September), and Quarter 4 (October, November, and December).

The outcome variables were the presence of tetracycline-resistant *Salmonella*, *Escherichia*, and *Campylobacter* isolates in retail meats and the presence of erythromycin- resistant *Campylobacter* isolates in retail meats. Tetracycline-resistant *Salmonella and Escherichia* isolates in retail meat were categorized based on the MIC breakpoint values of tetracycline (≥16 μg/ml). Similarly, the tetracycline-resistant *Campylobacter* in retail meats was categorized based on the MIC values of tetracycline (≥4 μg/ml). Erythromycin-resistant *Campylobacter* in retail meats was defined based on the MIC breakpoint values of erythromycin (≥8 μg/ml). The breakpoints were based on the 2021 NARMS Interpretive Criteria for Susceptibility Testing [53].

### Statistical analysis

Frequencies and percentages were used to summarize categorical predictor variables. Four mixed-effect logistic regression models were built, using a random intercept to control for state-level clustering for the tetracycline-resistant *Salmonella*, *Escherichia*, and *Campylobacter* isolates and erythromycin-resistant *Campylobacter* isolates in retail meats in this study.

For each model-building process, two steps were involved. In the first step, a univariable mixed effect logistic regression model using the state as a random intercept was fitted to assess the unadjusted associations between potential predictor variables and the outcome variable. We conducted the univariable analysis to investigate the association of the “year”, “years of sampling”, “VFD rule change”, meat type, quarter of year, and raising claims with the resistant outcome.

A relaxed p-value ≤ 0.2 [54–57] was used to identify predictors that were chosen for further examination in the multivariable mixed-effect logistic regression models in step two. To prevent the inclusion of collinear variables in the multivariable models, the pairwise collinearity of these variables was examined using Spearman’s rank correlation coefficient. If the correlation coefficient between two variables is ≥0.6 [58], only the variable with the highest odds ratio, the fewest missing observations in the initial screening, and biological plausibility would be included in the multivariable model. Categorical variables with more than two levels of categories were analyzed to evaluate pairwise differences using the Tukey-Kramer adjustment for multiple comparisons. Temporal graphs were generated in excel to visualize the temporal patterns of resistant outcomes by years of sampling. However, for the variables measuring similar characteristics (e.g., year, year of sampling, and VFD rule change), only one of the variables was used in model building, determined by the results of the univariable analysis.

In the second step, a multivariable mixed-effect logistic regression model (PROC GLIMMIX, SAS version 9.4) was built using a manual backward elimination method. A full model was first constructed by including all the screened variables and non-correlated as fixed factors, and subsequently, the state was fitted as a random effect and state as a random effect. We included the VFD rule change in every full multivariable model, regardless of the p-value obtained from the univariable analysis, as it was our primary exposure of interest for the analysis. Non-significant variables were removed through a manual backward elimination process. However, if the removal of a non-significant variable resulted in a substantial change (more than 20%) in the coefficients of any remaining variables in the model, it was considered a potential confounder and was retained in the final model [59]. Each final multivariable model was checked for possible multicollinearity using variance inflation factor (VIF) to avoid modeling issues associated with multicollinearity. If the VIF value exceeded 10, it indicated the of presence of multicollinearity [60]. The relevant pairwise significant interaction term was assessed in the final multivariable models [61]. For instance, the two-way interaction between VFD rule changes and the meat type was evaluated. For all final multivariable model results were presented as an odds ratio (OR) with a corresponding 95% confidence interval (CI), and p-values. A p-value ≤0.05 was considered statistically significant [62]. The overall assessment of the final multivariable model was done using the Akaike’s Information Criterion (AIC) [63]. The model with the lowest AIC values was considered as the best-fitting model.

## Results

The original dataset contained 342,041 records from 2002 to 2019. However, only 67,731 (19.8%) and 39,720 (11.6%) of these records had MIC values for the target antibiotics, tetracycline and erythromycin, respectively. Data regarding tetracycline-resistant *Salmonella* (N = 8,501), *Escherichia* (N = 20,283), *Campylobacter* (N = 9,682), and erythromycin-resistant *Campylobacter* (N = 10,446) from records of retail meats were included in the statistical analysis.

### Univariable mixed effect logistic regression results

Meat type, quarter of year, raising claims, and year were significantly associated with the occurrence of tetracycline-resistant *Salmonella* in retail meats (Table 1). Additionally, the estimated odds ratios from the years of sampling did not demonstrate a distinct linear pattern across all comparisons for tetracycline-resistant *Salmonella* in retail meats (S1 Table in S1 File). Furthermore, no linear trend was observed in the proportion of tetracycline-resistant *Salmonella* in retail meats based on our graphical analysis (S1 Fig in S1 File). Similarly, meat type, quarter of year, raising claims, year, and the VFD rule changes were significantly associated with tetracycline-resistant *Escherichia* in retail meats (Table 2). Additionally, the estimated odds ratios from the years of sampling did not demonstrate a distinct linear pattern across all comparison’s tetracycline-resistant *Escherichia* in retail meats (S2 Table in S1 File). Furthermore, no linear trend was observed the proportion of tetracycline-resistant *Escherichia* in retail meats based on our graphical analysis (S2 Fig in S1 File). Additionally, meat type, quarter, year, and VFD rule changes were significantly associated with tetracycline-resistant *Campylobacter* in retail meats (Table 3). However, there was no statistically significant association between raising claims and quarter year for tetracycline-resistant *Campylobacter* in retail meats (Table 3). In addition, the estimated odds ratios from the years of sampling did not demonstrate a distinct linear pattern across all comparison’s tetracycline-resistant *Campylobacter* in retail meats (S3 Table in S1 File). Furthermore, no linear trend was observed in the proportion of tetracycline-resistant *Campylobacter* in retail meats based on our graphical analysis (S3 Fig in S1 File). Lastly, meat type, year, and VFD rule changes were significantly associated with erythromycin-resistant *Campylobacter* in retail meats (Table 4). In addition, the estimated odds ratios from the years of sampling did not demonstrate a distinct linear pattern across all comparison’s erythromycin-resistant *Campylobacter* in retail meats (S4 Table in S1 File). Furthermore, no linear trend was observed in the proportion of erythromycin-resistant *Campylobacter* in retail meats based on our graphical analysis (S4 Fig in S1 File).

**Table 1:** Results of the univariable mixed-effect logistic regression model, using a random intercept to control for state-level clustering for tetracycline-resistant *Salmonella* in retail meats in the United States, 2002–2019.

Variable	Categories	Tetracycline-resistant		OR	95% CI	p-value
		Yes n (%)	No n (%)			
Year (n = 8,501)						<0.001
	2002	70 (45.75)	83 (54.25)	0.89	0.61, 1.29	0.554
	2003	76 (35.85)	136 (64.15)	0.60	0.43, 0.83	0.003
	2004	161 (49.69)	163 (50.31)	1.10	0.82, 1.46	0.502
	2005	146 (41.36)	207 (58.64)	0.83	0.63, 1.10	0.216
	2006	166 (49.11)	172 (50.89)	1.14	0.87, 1.51	0.327
	2007	178 (55.63)	142 (44.38)	1.55	1.17, 2.06	0.002
	2008	268 (54.58)	223 (45.42)	1.42	1.11, 1.83	0.005
	2009	298 (61.19)	189 (38.81)	1.77	1.37, 2.28	<0.001
	2010	218 (54.50)	182 (45.50)	1.37	1.05, 1.79	0.018
	2011	213 (59.66)	144 (40.34)	1.73	1.31, 2.28	<0.001
	2012	164 (46.20)	191 (53.80)	0.97	0.73, 1.27	0.828
	2013	183 (51.84)	170 (48.16)	1.26	0.96, 1.65	0.090
	2014	137 (52.29)	125 (47.71)	1.32	0.98, 1.78	0.063
	2015	185 (45.45)	222 (54.55)	0.95	0.73, 1.23	0.722
	2016	184 (43.91)	235 (56.09)	0.91	0.70, 1.18	0.509
	2017	257 (44.93)	315 (55.07)	Referent		
	2018	311 (44.05)	395 (55.95)	0.97	0.77, 1.21	0.795
	2019	1119 (56.17)	873 (43.83)	1.54	1.28, 1.87	<0.001
VFD rule changes (n = 8,501)						0.084
	Before VFD rule changes (2002–2016)	2647 (50.60)	2584 (49.40)	Referent		
	After VFD rule changes (2017–2019)	1687 (51.59)	1583(48.41)	1.09	0.98, 1.20	0.085
Meat type (n = 8,501)						<0.001
	Chicken breast	2382 (52.11)	2189(47.89)	1.00	0.92, 1.10	0.838
	Ground beef	61 (27.35)	162 (72.65)	0.36	0.26, 0.49	<0.001
	Ground turkey	1738 (51.79)	1618(48.21)	Referent		
	Pork chops	153 (43.59)	198 (56.41)	0.72	0.58, 0.91	0.006
Quarter of year (n = 8,501)						0.0187
	Quarter 1	1107 (51.54)	1041 (48.46)	1.14	1.00, 1.28	0.035
	Quarter 2	1139 (53.20)	1002 (46.80)	1.21	1.07, 1.36	0.002
	Quarter 3	1001 (48.06)	1082 (51.94)	Referent		
	Quarter 4	1087 (51.06)	1042 (48.94)	1.14	1.00, 1.28	0.035
Raising claims (n = 3,300)						0.0001
	Conventional	672 (47.93)	730 (52.07)	Referent		
	Without any antibiotics	1,027(54.11)	871(45.89)	1.33	1.15, 1.54	<0.001

OR = odds ratio; CI = confidence interval.

**Table 2:** Results of the univariable logistic regression models, using a random intercept to control for state-level clustering for tetracycline-resistant *Escherichia* in retail meats in the United States, 2002–2019.

Variable	Categories	Tetracycline-resistant		OR	95% CI	p-value
		Yes n (%)	No n (%)			
Year (n = 20,283)						<0.001
	2002	552 (51.83)	513 (48.17)	1.46	1.22, 1.74	<0.001
	2003	608 (48.33)	650 (51.67)	1.22	1.03, 1.45	0.016
	2004	678 (50.37)	668 (49.63)	1.31	1.11, 1.55	0.001
	2005	638 (48.70)	672 (51.30)	1.24	1.04, 1.46	0.012
	2006	679 (52.92)	604 (47.08)	1.45	1.22, 1.72	<0.001
	2007	505 (49.41)	517 (50.59)	1.23	1.03, 1.47	0.019
	2008	531 (52.99)	471 (47.01)	1.43	1.20, 1.71	<0.001
	2009	497 (48.97)	518 (51.03)	1.22	1.02, 1.46	0.024
	2010	537 (45.59)	641 (54.41)	1.07	0.90, 1.27	0.390
	2011	539 (50.37)	531 (49.63)	1.30	1.09, 1.55	0.003
	2012	577 (47.73)	632 (52.27)	1.17	0.99, 1.39	0.062
	2013	592 (50.64)	577 (49.36)	1.33	1.12, 1.58	0.001
	2014	572 (50.89)	552 (49.11)	1.35	1.13, 1.60	0.001
	2015	541 (50.66)	527 (49.34)	1.33	1.12, 1.59	0.001
	2016	445 (52.17)	408 (47.83)	1.42	1.18, 1.71	<0.001
	2017	585 (43.98)	745 (56.02)	Referent		
	2018	303 (45.50)	363 (54.50)	1.04	0.86, 1.25	0.680
	2019	604 (45.93)	711 (54.07)	1.06	0.90, 1.23	0.457
VFD rule changes (n = 20,283)						<0.001
	Before VFD rule changes (2002–2016)	8491 (50.03)	8481(49.97)	Referent		
	After VFD rule changes (2017–2019)	1492 (45.06)	1819 (54.94)	0.79	0.71, 0.87	<0.001
Meat type (n = 20,283)						<0.001
	Chicken breast	2489 (42.24)	3403(57.76)	0.23	0.22, 0.25	<0.001
	Ground beef	1014 (22.12)	3571(77.88)	0.09	0.08, 0.10	<0.001
	Ground turkey	4914(74.95)	1642 (25.05)	Referent		
	Pork chops	1566 (48.18)	1684 (51.82)	0.31	0.28, 0.33	<0.001
Quarter of year (n = 20,283)						<0.001
	Quarter 1	2720 (51.44)	2568 (48.56)	1.19	1.10, 1.29	<0.001
	Quarter 2	2588 (49.95)	2593(50.05)	1.12	1.03, 1.21	0.004
	Quarter 3	2315 (46.88)	2623(53.12)	Referent		
	Quarter 4	2360 (48.40)	2516 (51.60)	1.05	0.97, 1.14	0.200
Raising claims (n = 2,945)						<0.001
	Conventional	637 (49.57)	648 (50.43)	Referent		
	Without any antibiotics	687 (41.39)	973 (58.61)	0.71	0.61, 0.83	<0.001

OR = odds ratio; CI = confidence interval.

**Table 3:** Results of the univariable logistic regression models, using a random intercept to control for state-level clustering for tetracycline-resistant *Campylobacter* in retail meats in the United States, 2004–2019.

Variable	Categories	Tetracycline-resistant		OR	95% CI	p-value
		Yes n (%)	No n (%)			
Year (n = 9,698)						<0.001
	2004	354 (49.10)	367 (50.90)	1.17	0.94, 1.45	0.149
	2005	269 (46.86)	305 (53.14)	1.10	0.87, 1.39	0.386
	2006	289 (48.49)	307 (51.51)	1.11	0.88, 1.39	0.346
	2007	251 (48.46)	267 (51.54)	1.08	0.86, 1.37	0.480
	2008	279 (51.76)	260 (48.24)	1.34	1.06, 1.69	0.012
	2009	274 (45.29)	331 (54.71)	1.01	0.80, 1.26	0.916
	2010	200 (38.83)	315 (61.17)	0.77	0.61, 0.98	0.039
	2011	328 (51.74)	306 (48.26)	1.35	1.08, 1.68	0.007
	2012	330 (48.82)	346 (51.18)	1.23	0.99, 1.53	0.055
	2013	305 (47.81)	333 (52.19)	1.19	0.95, 1.48	0.112
	2014	243 (46.91)	275 (53.09)	1.20	0.94, 1.50	0.130
	2015	262 (45.02)	320 (54.98)	1.13	0.90, 1.41	0.264
	2016	270 (48.74)	284 (51.26)	1.21	0.97, 1.52	0.088
	2017	332 (41.97)	459 (58.03)	Referent		
	2018	205 (36.61)	355 (63.39)	0.75	0.60, 0.95	0.017
	2019	333 (49.19)	344 (50.81)	1.20	0.97, 1.48	0.091
VFD rule changes (n = 9,698)						0.008
	Before VFD rule changes (2004–2016)	3654 (47.64)	4016(52.36)	Referent		
	After VFD rule changes (2017–2019)	870 (42.90)	1158(57.10)	0.85	0.76, 0.96	0.008
Meat type (n = 9,698)						<0.001
	Chicken breast	4322 (45.90)	5095(54.10)	0.29	0.22, 0.38	<0.001
	Ground beef	1 (20.00)	4 (80.00)	0.10	0.01, 0.92	0.043
	Ground turkey	194 (73.21)	71 (26.79)	Referent		
	Pork chops	7 (63.64)	4 (36.36)	0.54	0.15, 1.95	0.355
Quarter of year (n = 9,698)						0.105
	Quarter 1	1060(44.31)	1332(55.69)	Referent		
	Quarter 2	1023(46.35)	1184(53.65)	1.07	0.95, 1.20	0.256
	Quarter 3	1178(47.56)	1299(52.44)	1.11	0.99, 1.25	0.062
	Quarter 4	1263(48.17)	1359(51.83)	1.14	1.02, 1.28	0.020
Raising claims (n = 2,276)						0.312
	Conventional	245 (43.91)	313 (56.09)	Referent		
	Without any antibiotics	808 (47.03)	910 (52.97)	1.11	0.90, 1.36	0.312

OR = odds ratio; CI = confidence interval.

**Table 4:** Results of the univariable logistic regression models, using a random intercept to control for state-level clustering for erythromycin-resistant *Campylobacter* in retail meats in the United States, 2004–2019.

Variable	Categories	Erythromycin-resistant		OR	95% CI	p-value
		Yes n (%)	No n (%)			
Year (n = 10,472)						<0.001
	2002	18 (6.06)	279 (93.94)	3.01	1.48, 6.11	0.002
	2003	16 (3.35)	461 (96.65)	2.00	0.97, 4.11	0.059
	2004	24 (3.33)	697 (96.67)	1.99	1.03, 3.84	0.040
	2005	19 (3.31)	555 (96.69)	1.93	0.97, 3.85	0.061
	2006	13 (2.18)	583 (97.82)	1.28	0.60, 2.73	0.511
	2007	14 (2.70)	504 (97.30)	1.59	0.76, 3.34	0.214
	2008	25 (4.64)	514 (95.36)	2.67	1.39, 5.12	0.003
	2009	12 (1.98)	593 (98.02)	0.85	0.39, 1.85	0.699
	2010	9 (1.75)	506 (98.25)	0.89	0.39, 2.06	0.803
	2011	14 (2.21)	620 (97.79)	1.04	0.50, 2.17	0.910
	2012	28 (4.14)	648 (95.86)	1.88	1.00, 3.53	0.049
	2013	27 (4.23)	611 (95.77)	2.31	1.23, 4.34	0.009
	2014	18 (3.47)	500 (96.53)	1.73	0.87, 3.44	0.115
	2015	34 (5.84)	548 (94.16)	2.96	1.61, 5.44	<0.001
	2016	16 (2.89)	538 (97.11)	1.48	0.73, 2.99	0.275
	2017	17 (2.15)	774 (97.85)	Referent		
	2018	5 (0.89)	555 (99.11)	0.38	0.13, 1.04	0.062
	2019	13 (1.92)	664 (98.08)	0.81	0.38, 1.72	0.602
VFD rule changes (n = 10,472)						<0.001
	Before VFD rule changes (2004–2016)	287 (3.40)	8,157(96.60)	Referent		
	After VFD rule changes (2017–2019)	35 (1.73)	1,993(98.27)	0.43	0.29, 0.63	<0.001
Meat type (n = 10,472)						<0.001
	Chicken breast	301 (2.96)	9,871(97.04)	0.64	0.37, 1.13	0.127
	Ground turkey	14 (5.11)	260 (94.89)	Referent		
	Ground beef & Pork	7 (26.92)	19 (73.08)	8.72	3.07, 24.75	<0.0001
Quarter of year (n = 10,472)						0.816
	Quarter 1	82 (3.20)	2,481(96.80)	1.14	0.83, 1.56	0.397
	Quarter 2	77 (3.22)	2,318(96.78)	1.14	0.83, 1.57	0.409
	Quarter 3	83 (3.09)	2,602(96.91)	1.10	0.80, 1.50	0.546
	Quarter 4	80 (2.83)	2,749(97.17)	Referent		
Raising claims (n = 22,76)						0.404
	Conventional	11 (1.97)	547 (98.03)	Referent		
	Without any antibiotics	24 (1.40)	1,694(98.60)	0.72	0.34, 1.53	0.405

OR = odds ratio; CI = confidence interval.

### Multivariable mixed effect logistic regression model results

The final model was fitted for tetracycline-resistant *Salmonella*, which included 8,501 records of retail meats (Table 5). Variables significantly associated with detecting tetracycline-resistant *Salmonella*—controlling for other variables—was meat type. No multicollinearity issue was found in the final model. However, there was a significant interaction between the VFD rule changes and meat type after controlling for all other variables in the model. The significant interaction between the VFD rule changes and meat type implies that the effect of the VFD rule changes on the odds of detecting tetracycline-resistant *Salmonella* was not the same across the meat types. For example, the odds of detecting tetracycline-resistant *Salmonella* were decreased by 44% in ground turkey following implementation of the VFD rule changes compared to ground turkey in the period prior to implementation (OR = 0.66, 95% CI: 0.56, 0.77; p<0.0001) (Table 5). In contrast, the odds of detecting tetracycline-resistant *Salmonella* were increased by 49% in the chicken breast following implementation of the VFD rule changes, compared to chicken breast in the period prior to implementation (OR = 1.49; 95% CI: 1.31, 1.69; p<0.0001) (Table 5).

**Table 5:** Final multivariable mixed-effect logistic regression model using a random intercept to control for state-level clustering for tetracycline-resistant *Salmonella* in retail meats (n = 8,501) in the United States, 2002–2019.

Variable	Categories	OR	95% CI	*P*-value
VFD rule changes				0.4146
	After the VFD rule changes (2017–2019) vs. Before the VFD rule changes (2002–2016))	0.91	0.72, 1.15	0.4146
Meat type				<0.0001
	Ground beef vs. Ground turkey	0.36	0.22, 0.63	<0.0001
	Chicken breast vs. Ground turkey	1.11	0.98 1.26	0.1462
	Pork chop vs. Ground turkey	0.75	0.55, 1.02	0.0804
VFD rule changes* Meat type				<0.0001
Ground beef				
	After VFD rule changes vs. before VFD rule changes	0.89	0.41 1.96	0.7741
Chicken breast				
	After VFD rule changes vs. before VFD rule changes	1.49	1.31, 1.69	<0.0001
Pork chop				
	After VFD rule changes vs. before VFD rule changes	0.77	0.49, 1.22	0.2708
Ground turkey				
	After VFD rule changes vs. before VFD rule changes	0.66	0.56, 0.77	0.56,	0.77	<0.0001
0.56,	0.77
Before VFD rule changes				
	Ground beef vs. Ground turkey	0.32	0.21, 0.50	<0.0001
	Chicken breast vs. Ground turkey	0.74	0.63, 0.86	<0.0001
	Pork chop vs. Ground turkey	0.69	0.48, 0.99	0.0385
	Chicken breast vs. Ground beef	2.29	1.47, 3.55	<0.0001
	Chicken breast vs. Pork chop	1.07	0.75, 1.52	0.9665
	Ground beef vs. Pork chop	0.47	0.27, 0.81	0.27,	0.81	0.0019
0.27,	0.81
After the VFD rule changes				
	Ground beef vs. Ground turkey	0.44	0.17, 1.13	0.1133
	Chicken breast vs. Ground turkey	1.67	1.37, 2.03	<0.0001
	Pork chop vs. Ground turkey	0.81	0.49, 1.36	0.7242
	Chicken breast vs. Ground beef	3.83	1.49, 9.85	0.0015
	Chicken breast vs. Pork chop	2.06	1.24, 3.41	0.0013
	Ground beef vs. Pork chop	0.54	0.19, 1.55	0.4327

OR—Odds ratio; CI—Confidence interval.

The final model was fitted for tetracycline-resistant *Escherichia*, which included 20,283 records of retail meat (Table 6). Variables significantly associated with detecting tetracycline-resistant *Escherichia*—controlling for other variables—were the VFD rule changes, meat type, and sampling quarter. No multicollinearity issue was found in the final model. However, there was a significant interaction between the VFD rule changes and meat type after controlling for all other variables in the model. The significant interaction between the VFD rule changes and meat type implies that the effect of the VFD rule changes on the odds of detecting tetracycline-resistant *Escherichia* was not the same across the meat types. For example, the odds of detecting tetracycline-resistant *Escherichia* were decreased by 40% in the chicken breast following the implementation of the VFD rule changes compared to chicken breast in the period prior to implementation (OR = 0.60, 95% CI: 0.50, 0.72; p<0.0001) (Table 6). Likewise, the odds of detecting tetracycline-resistant *Escherichia* were decreased by 44% in ground turkey following implementation of the VFD rule changes compared to ground turkey in the period prior to implementation (OR = 0.56, 95% CI: 0.48, 0.65; p<0.0001) (Table 6). After the VFD rule changes, there was no significant difference in the odds of detecting tetracycline-resistant *Escherichia* in ground beef and pork chop compared to before the VFD rule changes (Table 6). Considering quarter of year, the odds of detecting tetracycline-resistant *Escherichia* in Quarter 1 and Quarter 2 were 22% (OR = 1.22, 95% CI: 1.09, 1.36); p<0.0001) and 12% (OR = 1.12, 95% CI: 1.00, 1.26, p = 0.008) higher, compared to Quarter 3 (Table 6).

**Table 6:** Final multivariable mixed-effect logistic regression model using a random intercept to control for state-level clustering for tetracycline-resistant *Escherichia* in retail meats (n = 20,283) in the United States, 2002–2019.

Variable	Categories	OR	95% CI	*P*-value
VFD rule changes				<0.0001
	After the VFD rule changes (2017–2019) vs. Before the VFD rule changes (2002–2016))	0.72	0.64, 0.81	<0.0001
Meat type				<0.0001
	Ground beef vs. Ground turkey	0.11	0.09, 0.13	<0.0001
	Chicken breast vs. Ground turkey	0.24	0.21, 0.27	<0.0001
	Pork chop vs. Ground turkey	0.36	0.31, 0.42	<0.0001
Sampling quarter				<0.0001
	Quarter 1vs. Quarter 3	1.22	1.09, 1.36	<0.0001
	Quarter 2 vs. Quarter 3	1.12	1.00, 1.26	0.0399
	Quarter 4 vs. Quarter 3	1.04	0.93, 1.16	0.8419
VFD rule changes* Meat type				<0.0001
Ground beef				
	After VFD rule changes vs. before VFD rule changes	0.89	0.72, 1.10	0.2742
Chicken breast				
	After VFD rule changes vs. before VFD rule changes	0.60	0.50, 0.72	<0.0001
Pork chop				
	After VFD rule changes vs. before VFD rule changes	0.90	0.74, 1.10	0.3091
Ground turkey				
	After VFD rule changes vs. before VFD rule changes	0.56	0.48, 0.65	<0.0001
Before VFD rule changes				
	Ground beef vs. Ground turkey	0.09	0.07, 0.10	<0.0001
	Chicken breast vs. Ground turkey	0.23	0.20, 0.25	<0.0001
	Pork chop vs. Ground turkey	0.28	0.25, 0.32	<0.0001
	Chicken breast vs. Ground beef	2.68	2.37, 3.03	<0.0001
	Chicken breast vs. Pork chop	0.80	0.71, 0.91	<0.0001
	Ground beef vs. Pork chop	0.30	0.26, 0.35	<0.0001
After the VFD rule changes				
	Ground beef vs. Ground turkey	0.14	0.10 0.18	<0.0001
	Chicken breast vs. Ground turkey	0.25	0.19, 0.32	<0.0001
	Pork chop vs. Ground turkey	0.46	0.35, 0.60	<0.0001
	Chicken breast vs. Ground beef	1.81	1.33, 2.47	<0.0001
	Chicken breast vs. Pork chop	0.53	0.40, 0.71	<0.0001
	Ground beef vs. Pork chop	0.29	0.21, 0.41	<0.0001

OR—Odds ratio; CI—Confidence interval.

The final model was fitted for tetracycline-resistant *Campylobacter*, which included 9,682 records of retail meats (Table 7). Variables significantly associated with detecting tetracycline-resistant *Campylobacter*—controlling for other variables—were the VFD rule changes and meat type. No multicollinearity issue was found in the final model. However, there was a significant interaction between the VFD rule changes and meat type after controlling for all other variables in the model. The significant interaction between the VFD rule changes and meat type implies that the effect of the VFD rule changes on the odds of detecting tetracycline-resistant *Campylobacter* was not the same across the meat types. For example, the odds of detecting tetracycline-resistant *Campylobacter* were decreased by 11% in the chicken breast following implementation of the VFD rule changes, compared to chicken breast in the period prior to implementation (OR = 0.89, 95% CI: 0.79, 0.99; p = 0.0370) (Table 6). Likewise, the odds of detecting tetracycline-resistant *Campylobacter* were decreased by 67% in ground turkey following implementation of the VFD rule changes, compared to ground turkey in the period prior to implementation (OR = 0.33, 95% CI: 0.17, 0.66; p = 0.0017) (Table 7).

**Table 7:** Final multivariable mixed-effect logistic regression model using a random intercept to control for state-level clustering for tetracycline-resistant *Campylobacter* in retail meats (n = 9,682) in the United States, 2002–2019.

Variable	Categories	OR	95% CI	*P*-value
VFD rule changes				0.0007
	After the VFD rule changes (2017–2019) vs. Before the VFD rule changes (2002–2016))	0.54	0.38, 0.77	0.0007
Meat type				<0.0001
	Chicken breast vs. Ground turkey	0.40	0.28, 0.56	<0.0001
VFD rule changes* Meat type				0.0058
Chicken breast				
	After VFD rule changes vs. before VFD rule changes	0.89	0.79, 0.99	0.0370
Ground turkey				
	After VFD rule changes vs. before VFD rule changes	0.33	0.17, 0.66	0.0017
Before VFD rule changes				
	Chicken breast vs. Ground turkey	0.243	0.18, 0.34	<0.0001
After the VFD rule changes				
	Chicken breast vs. Ground turkey	0.65	0.35, 1.20	0.1685

OR—Odds ratio; CI—Confidence interval.

The final model was fitted for erythromycin-resistant *Campylobacter*, which included 10,446 records of retail meats (Table 8). Variables significantly associated with detecting erythromycin-resistant *Campylobacter*—controlling for other variables—was meat type. No multicollinearity issue was found in the final model. However, there was a significant interaction between the VFD rule changes and meat type after controlling for all other variables in the model. The significant interaction between the VFD rule changes and meat type implies that the effect of the VFD rule changes on the odds of detecting erythromycin-resistant *Campylobacter* were not the same across the meat types. For example, the odds of detecting erythromycin-resistant *Campylobacter* were decreased by 57% in the chicken breast following implementation of the VFD rule changes, compared to chicken breast in the period prior to implementation (OR = 0.43, 95% CI: 0.29, 0.64; p<0.0001). In contrast, the odds of detecting erythromycin-resistant *Campylobacter* were 4.63 times higher in ground turkey following implementation of the VFD rule changes compared to ground turkey in the period prior to implementation (OR = 4.63; 95% CI: 1.48, 14.44; p = 0.0084) (Table 8).

**Table 8:** Final multivariable mixed-effect logistic regression model using a random intercept to control for state-level clustering for erythromycin-resistant *Campylobacter* in retail meats (n = 10,446) in the United States, 2002–2019.

Variable	Categories	OR	95% CI	*P*-value
VFD rule changes				0.2584
	After the VFD rule changes (2017–2019) vs. Before the VFD rule changes (2002–2016))	1.41	0.78, 2.58	0.2584
Meat type				<0.0001
	Chicken breast vs. Ground turkey	0.30	0.16, 0.54	<0.0001
VFD rule changes* Meat type				0.0001
Chicken breast				
	After VFD rule changes vs. before VFD rule changes	0.43	0.29, 0.64	<0.0001
Ground turkey				
	After VFD rule changes vs. before VFD rule changes	4.63	1.48, 14.44	0.0084
Before VFD rule changes				
	Chicken breast vs. Ground turkey	0.97	0.47, 1.99	0.9286
After the VFD rule changes				
	Chicken breast vs. Ground turkey	0.09	0.04, 0.24	<0.0001

OR—Odds ratio; CI—Confidence interval.

## Discussion

The VFD rules changes were implemented in 2017 to reduce the use of medically important antibiotics in food-producing animals. These rule changes are crucial in decreasing the amount of medically important antibiotics used and, consequently, reducing the development of antibiotic-resistant bacteria in food-producing animals and their products in the U.S. This current investigation sought to determine the association between VFD rule changes and the occurrence of tetracycline and erythromycin-resistant bacteria isolated from retail meats obtained under the NARMS in the U.S., after controlling for other variables. After controlling for other variables in the multivariable models, there was a significant interaction between the VFD rule changes and meat type on the occurrence of tetracycline-resistant *Salmonella* in retail meats (Table 5). Additionally, after controlling for other variables in the multivariable models, there was a significant interaction between the VFD rule changes and meat type on the occurrence of tetracycline-resistant *Escherichia* in retail meats (Table 6). Likewise, after controlling for other variables in the multivariable models, there was a significant interaction between the VFD rule changes and meat type on the occurrence of tetracycline-resistant *Campylobacter* in retail meats (Table 7). Moreover, after controlling for other variables in the multivariable models, there was a significant interaction between the VFD rule changes and meat type on the occurrence of erythromycin-resistant *Campylobacter* in retail meats (Table 8). The significant interaction between the VFD rule changes and meat type implies that the effect of the VFD rule changes on the odds of detecting antibiotic-resistant bacteria were not the same across the meat types.

The findings of this study reveal that the implementation of VFD rule changes in the U.S. resulted in a significant reduction in the odds of detecting tetracycline-resistant *Escherichia*, *Campylobacter*, and erythromycin-resistant *Campylobacter* isolated from chicken breast. Also, our data revealed significant reduction in the odds of detecting tetracycline-resistant *Salmonella*, *Escherichia*, and *Campylobacter* isolated from ground turkey. To our knowledge, this report is the first to provide quantitative evidence of the association between changes in VFD rules changes and the odds of detecting tetracycline-resistant *Escherichia*, *Campylobacter*, and erythromycin-resistant *Campylobacter* isolated from chicken breast, as well as tetracycline-resistant *Salmonella*, *Escherichia*, and *Campylobacter* in ground turkey in the U.S.

Multiple factors could explain the decreased odds of detecting tetracycline-resistant *Escherichia* (40%) and *Campylobacter* (11%) isolated from chicken breast compared to the period before implementation. One is the VFD rule changes potentially decreased the use of tetracycline in chicken production, leading to a decrease in the occurrence of tetracycline-resistant bacteria in chicken breast in the U.S. Reduced use of tetracycline may have reduced the selective pressure for the emergence of tetracycline-resistant bacteria in food animals, including chickens [13,15]. The U.S. FDA’s recent report indicates that tetracycline sales decreased in chicken production after the VFD rule changes were implemented [11]. Recent data on on-farm antimicrobial use in the U.S. broiler chicken industry shows a substantial decline in the utilization of medically important in-feed and water-soluble tetracycline since 2017 [64]. Similarly, data on on-farm antimicrobial use in U.S. turkey production demonstrates a substantial reduction in the usage of medically important in-feed and water-soluble tetracycline in 2017 [65]. The reduction of antimicrobial use in food-producing animals is associated with a reduction in the occurrence of antibiotic-resistant bacteria originating from food animals [15]. An observational study conducted by Stuart B. Levy et al. [66] demonstrated that six months after the removal of tetracycline-supplemented feed from a chicken farm, there was a lower frequency of tetracycline-resistant *Escherichia* isolates compared to before the removal of the tetracycline-supplemented feed in the farm. There is also evidence that a reduction in antimicrobial use is associated with reduced occurrence of antimicrobial-resistant bacteria isolates from humans [21,56]. In addition, chicken production type and management practices could be associated with decreased odds of detecting tetracycline-resistant *Escherichia* and *Campylobacter*. For instance, extensive and comprehensive cleaning and hygiene practices in chicken farms could reduce the reservoirs of resistant bacteria [67] that may have occurred on dirty floors and fomites within the chicken houses [68]. Also, improved feeding systems, such as providing safe and antibiotic-free feed and water in chicken production, could reduce the risk of the occurrence of resistant bacteria [67] in chicken meats. Furthermore, the organic food industry is rapidly growing in the U.S., with organic meat and poultry sales increasing by 2.5% and 4.7%, respectively, in 2021 [69]. Transitioning from conventional chicken production to organic chicken production, which involves the elimination of antibiotic use, can significantly reduce the occurrence of antibiotic-resistant bacteria in chicken meat in the U.S. There is evidence that the prevalence of antibiotic-resistant *Enterococcus* significantly decreased in poultry farms in the U.S. which transitioned from conventional farms to organic farming practices [70].

Another explanation could be that the VFD rule changes may have improved veterinary-client-patient relationships, resulting in better oversight of antimicrobial usage in chicken production by licensed veterinarians. For instance, following the implementation of VFD rule changes in the U.S., administering medically important antimicrobials to food animals via feed or drinking water has been brought under licensed veterinarian supervision [71]. Additionally, it is possible that the VFD rule changes led to increased awareness and adoption of responsible antimicrobial use practices among both chicken producers and veterinarians, resulting in more judicious use of antimicrobials to help mitigate the development of resistance to antimicrobials [72,73]. Also, it is possible that the reduction in antimicrobial-resistant bacteria from retail chicken meats observed after the implementation of VFD rule changes may be due to factors not directly related to the rule changes—such as natural variations in resistance patterns in meat-associated bacterial population over time or improved hygiene practices during meat processing at slaughterhouses, in storing, or handling. Overall, this study demonstrates that the VFD rule changes were beneficial in reducing the occurrence of tetracycline-resistant *Escherichia* and *Campylobacter* in chicken breasts in the U.S.

However, the multivariable logistic regression analysis showed that the odds of detecting tetracycline-resistant *Salmonella* were significantly increased in the chicken breast following the VFD rule changes compared to the pre-VFD rule changes period. The findings of our study is consistent with previous research that has reported a higher level of tetracycline-resistant *Salmonella* in chicken breast compared to ground turkey in California in 2018 [57]. The exact cause of this outcome remains unknown. However, it is possible that the composition of *Salmonella* serotypes within chicken production may vary over time [74], or there could be other unidentified factors that explain our study’s findings. Control of tetracycline-resistant *Salmonella* in chicken meat is difficult as chickens can serve as perpetual vectors and reservoirs without exhibiting symptoms [57]. Tetracycline-resistant *Salmonella* can contaminate the chicken farm environment and potentially spread the bacteria by infected chicken and contaminated eggs [74]. It is important to conduct further investigations to explore potential risk factors associated with an increased likelihood of tetracycline-resistant *Salmonella* in chicken meat.

Our research shows that there was no significant change in the likelihood of detecting tetracycline-resistant *Salmonella* and *Escherichia* in ground beef and pork chops after the VFD rule changes were implemented in 2017, compared to before the changes. Our study’s results align with recent research that found no significant change in the prevalence of tetracycline-resistant *Escherichia* in cecal samples of swine at slaughter in the U.S. between 2013 and 2019 [75]. There is no systematic collection of medically important antibiotic consumption data at the farm-level in the U.S. [71]. Such data could provide important information to explain the observed findings. However, the FDA’s medically important antibiotic drug and species-specific sales data between 2016 and 2019 could serve as a proxy for antibiotic consumption data at the farm-level in the U.S. [11]. The data shows that tetracycline sales were highest in 2016 for both cattle (2,840,519 kilograms) and swine (2,520,680 kilograms) production. Tetracycline sales sharply decreased for cattle (1,560,542 kilograms) and swine (1,579,145 kilograms) production in 2017 when the VFD rule changes were implemented. However, tetracycline sales increased in both cattle and swine production in 2018 and 2019 in the U.S. [11]. This pattern of tetracycline sales suggests that there was no consistent reduction in tetracycline consumption in cattle and swine production following the implementation of the VFD rule changes measures in the U.S. Furthermore, the increased usage of this drug for therapeutic purposes could also contribute to the rising sales of tetracycline following the implementation of the VFD rule changes. It is important to conduct further investigations to explore potential factors associated with the increased usage of tetracycline in cattle and swine production just one year after implementing the VFD rule changes.

The current study also shows that there were lower odds of detecting erythromycin-resistant *Campylobacter* in chicken breast (57%), following the VFD rule changes implementation period, compared to the period prior to implementation. These observed lower odds of detecting erythromycin-resistant *Campylobacter* in chicken breast may be due to declines in the frequency of usage of erythromycin following the implementation of VFD rule changes. This decrease in usage could result in lower selective pressure [72,73]. The U.S. FDA antimicrobials sold data indicates that the sale of macrolides, including erythromycin, sharply decreased in chicken production following the 2017 VFD rule changes [11]. Alternative explanations may include improved farming management and biosecurity practices, resulting in a decreased occurrence of bacterial disease (respiratory disease, infectious sinusitis, air sacculitis), and, thus, a reduction of the need for macrolide (erythromycin, tylosin) usage in chicken production. There is evidence from other studies that reducing or restricting antimicrobial use in food-producing animals reduces the occurrence of antimicrobial-resistant bacteria by decreasing selective pressure [72,73] on the bacteria originating from the food animals [15,76]. Likewise, a low prevalence of ciprofloxacin-resistant *Campylobacter* in Louisiana retail chickens was observed after the enrofloxacin ban [77]. In human studies, evidence of low use of azithromycin (macrolide) during summer months was found to be associated with decreased prevalence of resistant pneumococci among children [18,78]. The findings of our work show that implementing the VFD rule changes has had a significant effect on the occurrence of erythromycin-resistant *Campylobacter* in chicken breast in the U.S.

However, the odds of detecting erythromycin-resistant *Campylobacter* were significantly increased in ground turkey following the implementation of the VFD rule changes compared to the pre-VFD rule changes period. There was no systematic collection of medically important antibiotic consumption data at the turkey farm level in the U.S. [71]. Such data could provide important information to explain this finding. However, the FDA’s medically important antibiotic drug and species-specific sales data between 2016 and 2019 could serve as a proxy for antibiotic consumption data at the turkey farm-level in the U.S. The data shows a sharp increase in sales of macrolide, including erythromycin, in 2017 (1307 kilograms) when the VFD rule changes were implemented [11]. An increasing trend in macrolide sales was observed in 2018 (1653 kilograms) and 2019 (1944 kilograms) in the U.S. [11]. This sales pattern suggests an increasing consumption of macrolide, including erythromycin/tylosin, in turkey production following the implementation of the VFD rule changes in the U.S., which can create selective pressure in developing erythromycin-resistant bacteria. Previous research identified erythromycin-resistant *Campylobacter* in commercial turkey farms and at slaughterhouses in Ohio, USA [79]. There is evidence of an association between antimicrobial usage and antimicrobial-resistant bacteria isolated from fecal samples in turkey farms in European countries [80]. Other possible reasons may be the presence of poor hygiene, inadequate flock health management, and intensive farming conditions at turkey farms. These factors can contribute to a higher prevalence of infectious diseases such as respiratory disease/infectious sinusitis/air sacculitis in turkeys. These conditions often require treatment with antimicrobial drugs from the macrolide group. The usage of antibiotics can increase the selection pressure for erythromycin-resistant bacteria among turkeys [81]. As a result, there is an increased likelihood of erythromycin-resistant bacteria-infected turkeys entering slaughterhouse establishments. Additionally, suppose turkeys are exposed to feed or water contaminated with erythromycin-resistant bacteria or genes. In that case, they can become carriers of these resistant bacteria. Furthermore, improper sanitary and cleanliness of slaughterhouses and fecal contamination during slaughtering processing [82,83] can result in resistant-bacteria cross-contamination in turkey meat. Evidence of improper scalding or scalding processes without scalding water temperature control was significantly associated with bacteria contamination of chicken meat in slaughterhouses [84]. The U.S. is the world’s largest producer of turkey and turkey meat. In 2019, approximately 229 million turkeys were raised in the U.S., resulting in a total live weight production of 7.4 billion pounds. It is important to conduct further investigations at the turkey farm-level to explore potential factors associated with the emergence of erythromycin-resistant *Campylobacter* within U.S. turkey production following the implementation of the VFD rule changes.

While this study provides valuable insights into the reduction of tetracycline-resistant *Escherichia*, *Campylobacter*, and erythromycin-resistant *Campylobacter* in chicken breast, as well as tetracycline-resistant *Salmonella*, *Escherichia*, and *Campylobacter* in ground turkey, it is important to note that these findings may not apply to all chicken and turkey retail meats sold in the U.S. This limitation arises from the sampling strategy employed during the study period (2002–2019), which involved convenience sampling [85]. The utilization of convenience sampling could potentially introduce sample selection bias.

To analyze the occurrence of antibiotic-resistant bacteria in retail meats, we initially examined both the individual year and three-year trend using a univariable mixed effect logistic regression model. Additionally, we visually assessed the linear trend of the proportion of antibiotic-resistant bacteria in retail meats at three-year intervals. However, the estimated odds ratios from both the individual year and three-year wise analyses did not demonstrate a consistent linear pattern across all comparisons (S1, S2, S3 and S4 Tables in S1 File). Furthermore, no linear trend of the proportion of antibiotic-resistant bacteria in retail meats was observed based on our graphical analysis (S1, S2, S3 and S4 Figs in S1 File). These non-linear relationships suggest that the impact of individual years or three-year intervals on antibiotic-resistant bacteria in retail meats vary across different points of comparison. Considering these observations, we chose to analyze the variable “VFD rule change” as a reasonable approach. We categorized data into two groups: “before VFD rule change (2002–2016)” and “after VFD rule change (2017–2019)”. Our research question aimed to investigate significant differences in antibiotic-resistant bacteria in retail meats, and the “VFD rule change” variable allowed us to examine the overall effect of the period after the implementation of VFD rule changes compared to the reference period (pre-VFD period: 2002–2016). Furthermore, collapsing these years into binary variable increased the sample size of the category, thereby improving the statistical power of our analysis. Additionally, we employed a mixed-effect logistic regression model to address the clustering effect of state-level during the analysis to avoid biased estimates [86].

The findings of this study suggest that the implementation of VFD rule changes had a positive impact on reducing the presence of tetracycline and erythromycin-resistant bacteria (*Escherichia and Campylobacter*) in retail meats, specifically chicken breast and ground turkey. The implementation of VFD rule changes has likely contributed to a decrease in antibiotic-resistant bacteria isolates found in retail meats by reducing the usage of antibiotics in food animals. These findings highlight the importance of continued efforts to promote the judicious use of medically important antimicrobials in food-producing animals to combat the emergence and spreading of antimicrobial-resistant bacteria. Further research is necessary covering a more extended period after the implementation of VFD rule changes could more comprehensively evaluate the effectiveness of the VFD rule changes on the occurrence of bacteria resistant to medically-important antimicrobials in retail meats in the U.S. Additionally, it is crucial to assess the impact of the implementation of the VFD rule changes on human health in the U.S.

## Supporting information

S1 File Contains all the supporting tables and figures. (DOCX)
