# Supplementary material for: Effect of veterinary feed directive rule changes on tetracycline-resistant and erythromycin-resistant bacteria (Salmonella, Escherichia, and Campylobacter) in retail meats in the United States
Source: PLoS One. 2023 Aug 3;18(8):e0289208. doi: 10.1371/journal.pone.0289208 (PMC10399851; doi:10.1371/journal.pone.0289208)

PONE-D-23-11107R1

Effect of veterinary feed directive rule changes on tetracycline-resistant and erythromycin-resistant bacteria (*Salmonella*, *Escherichia*, and *Campylobacter*) in retail meats in the United States

**S1 Table. Univariable mixed-effect logistic regression of association between years of sampling and tetracycline-resistant *Salmonella* in retail meats in the United States**

| **Variable** | **Categories** | **OR** | **95% CI** | ***P*-value** |
| --- | --- | --- | --- | --- |
| **Years of sampling**  (n= 8501) |  |  |  | <0.0001 |
|  | 2002-2004 vs. 2017-2019 | 0.68 | 0.52, 0.88 | 0.0002 |
|  | 2005-2007 vs. 2017-2019 | 0.87 | 0.69, 1.08 | 0.4621 |
|  | 2008-2010 vs. 2017-2019 | 1.17 | 0.96, 1.43 | 0.205 |
|  | 2011-2013 vs. 2017-2019 | 0.99 | 0.80, 1.23 | 1 |
|  | 2014-2016 vs. 2017-2019 | 0.79 | 0.64, 0.97 | 0.014 |
|  | 2002-2004 vs. 2005-2007 | 0.78 | 0.58, 1.04 | 0.1237 |
|  | 2002-2004 vs. 2008-2010 | 0.58 | 0.44, 0.76 | <0.0001 |
|  | 2002-2004 vs. 2011-2013 | 0.68 | 0.51, 0.91 | 0.0021 |
|  | 2002-2004 vs. 2014-2016 | 0.86 | 0.64, 1.15 | 0.6604 |
|  | 2005-2007 vs. 2008-2010 | 0.74 | 0.58, 0.95 | 0.0062 |
|  | 2005-2007 vs. 2011-2013 | 0.88 | 0.68, 1.13 | 0.6955 |
|  | 2005-2007 vs. 2014-2016 | 1.11 | 0.86, 1.43 | 0.8701 |
|  | 2008-2010 vs. 2011-2013 | 1.18 | 0.93, 1.50 | 0.3355 |
|  | 2008-2010 vs. 2014-2016 | 1.49 | 1.18, 1.89 | <0.0001 |
|  | 2011-2013 vs. 2014-2016 | 1.26 | 0.98, 1.62 | 0.088 |

OR — Odds ratio; CI — Confidence interval

**S2 Table. Univariable mixed-effect logistic regression of association between years of sampling and tetracycline-resistant *Escherichia* in retail meats in the United States**

| **Variable** | **Categories** | **OR** | **95% CI** | ***P*-value** |
| --- | --- | --- | --- | --- |
| **Years of sampling** (n=20283) |  |  |  | 0.0001 |
|  | 2002-2004 vs. 2017-2019 | 1.29 | 1.09, 1.53 | 0.0004 |
|  | 2005-2007 vs. 2017-2019 | 1.27 | 1.07, 1.51 | 0.0008 |
|  | 2008-2010 vs. 2017-2019 | 1.19 | 1.00, 1.42 | 0.0473 |
|  | 2011-2013 vs. 2017-2019 | 1.23 | 1.04, 1.46 | 0.0078 |
|  | 2014-2016 vs. 2017-2019 | 1.33 | 1.11, 1.58 | <0.0001 |
|  | 2002-2004 vs. 2005-2007 | 1.01 | 0.88, 1.16 | 0.9999 |
|  | 2002-2004 vs. 2008-2010 | 1.08 | 0.94, 1.24 | 0.6314 |
|  | 2002-2004 vs. 2011-2013 | 1.05 | 0.91, 1.20 | 0.9343 |
|  | 2002-2004 vs. 2014-2016 | 0.97 | 0.84, 1.12 | 0.9904 |
|  | 2005-2007 vs. 2008-2010 | 1.07 | 0.93, 1.23 | 0.7672 |
|  | 2005-2007 vs. 2011-2013 | 1.03 | 0.90 1.19 | 0.9812 |
|  | 2005-2007 vs. 2014-2016 | 0.96 | 0.83, 1.10 | 0.9602 |
|  | 2008-2010 vs. 2011-2013 | 0.97 | 0.84, 1.12 | 0.9887 |
|  | 2008-2010 vs. 2014-2016 | 0.90 | 0.78, 1.04 | 0.2970 |
|  | 2011-2013 vs. 2014-2016 | 0.93 | 0.81, 1.07 | 0.6596 |

OR — Odds ratio; CI — Confidence interval

**S3 Table. Univariable mixed-effect logistic regression of association between years of sampling and tetracycline-resistant *Campylobacter* in retail meats in the United States**

| **Variable** | **Categories** | **OR** | **95% CI** | ***P*-value** |
| --- | --- | --- | --- | --- |
| **Years of sampling**  (n= 9698) |  |  |  | 0.0049 |
|  | 2002-2004 vs. 2017-2019 | 1.18 | 0.90, 1.55 | 0.4947 |
|  | 2005-2007 vs. 2017-2019 | 1.11 | 0.90, 1.38 | 0.7184 |
|  | 2008-2010 vs. 2017-2019 | 1.03 | 0.84, 1.28 | 0.9976 |
|  | 2011-2013 vs. 2017-2019 | 1.27 | 1.04, 1.55 | 0.0085 |
|  | 2014-2016 vs. 2017-2019 | 1.20 | 0.97, 1.47 | 0.1339 |
|  | 2002-2004 vs. 2005-2007 | 1.06 | 0.82, 1.38 | 0.9863 |
|  | 2002-2004 vs. 2008-2010 | 1.14 | 0.88, 1.48 | 0.6983 |
|  | 2002-2004 vs. 2011-2013 | 0.93 | 0.72, 1.20 | 0.9661 |
|  | 2002-2004 vs. 2014-2016 | 0.99 | 0.76, 1.29 | 1.0000 |
|  | 2005-2007 vs. 2008-2010 | 1.08 | 0.88, 1.32 | 0.9106 |
|  | 2005-2007 vs. 2011-2013 | 0.88 | 0.72, 1.07 | 0.3881 |
|  | 2005-2007 vs. 2014-2016 | 0.93 | 0.76, 1.15 | 0.9260 |
|  | 2008-2010 vs. 2011-2013 | 0.82 | 0.67, 0.99 | 0.0329 |
|  | 2008-2010 vs. 2014-2016 | 0.87 | 0.70, 1.07 | 0.3618 |
|  | 2011-2013 vs. 2014-2016 | 1.06 | 0.87, 1.29 | 0.9510 |

OR — Odds ratio; CI — Confidence interval

**S4 Table. Univariable mixed-effect logistic regression of association between years of sampling and erythromycin-resistant *Campylobacter* in retail meats in the United States**

| **Variable** | **Categories** | **OR** | **95% CI** | ***P*-value** |
| --- | --- | --- | --- | --- |
| **Years of sampling** (n= 10472) |  |  |  | 0.0004 |
|  | 2002-2004 vs. 2017-2019 | 2.29 | 1.23, 4.27 | 0.0021 |
|  | 2005-2007 vs. 2017-2019 | 1.59 | 0.83, 3.05 | 0.3213 |
|  | 2008-2010 vs. 2017-2019 | 1.62 | 0.85, 3.11 | 0.2761 |
|  | 2011-2013 vs. 2017-2019 | 2.09 | 1.14, 3.81 | 0.0068 |
|  | 2014-2016 vs. 2017-2019 | 2.43 | 1.33, 4.46 | 0.0004 |
|  | 2002-2004 vs. 2005-2007 | 1.44 | 0.81, 2.56 | 0.4630 |
|  | 2002-2004 vs. 2008-2010 | 1.41 | 0.79, 2.52 | 0.5262 |
|  | 2002-2004 vs. 2011-2013 | 1.09 | 0.65, 1.85 | 0.9957 |
|  | 2002-2004 vs. 2014-2016 | 0.94 | 0.56, 1.59 | 0.9995 |
|  | 2005-2007 vs. 2008-2010 | 0.98 | 0.53, 1.80 | 1.0000 |
|  | 2005-2007 vs. 2011-2013 | 0.76 | 0.44, 1.33 | 0.7319 |
|  | 2005-2007 vs. 2014-2016 | 0.65 | 0.37, 1.14 | 0.2511 |
|  | 2008-2010 vs. 2011-2013 | 0.78 | 0.45, 1.36 | 0.7893 |
|  | 2008-2010 vs. 2014-2016 | 0.67 | 0.38, 1.17 | 0.3016 |
|  | 2011-2013 vs. 2014-2016 | 0.86 | 0.52, 1.42 | 0.9519 |

OR — Odds ratio; CI — Confidence interval


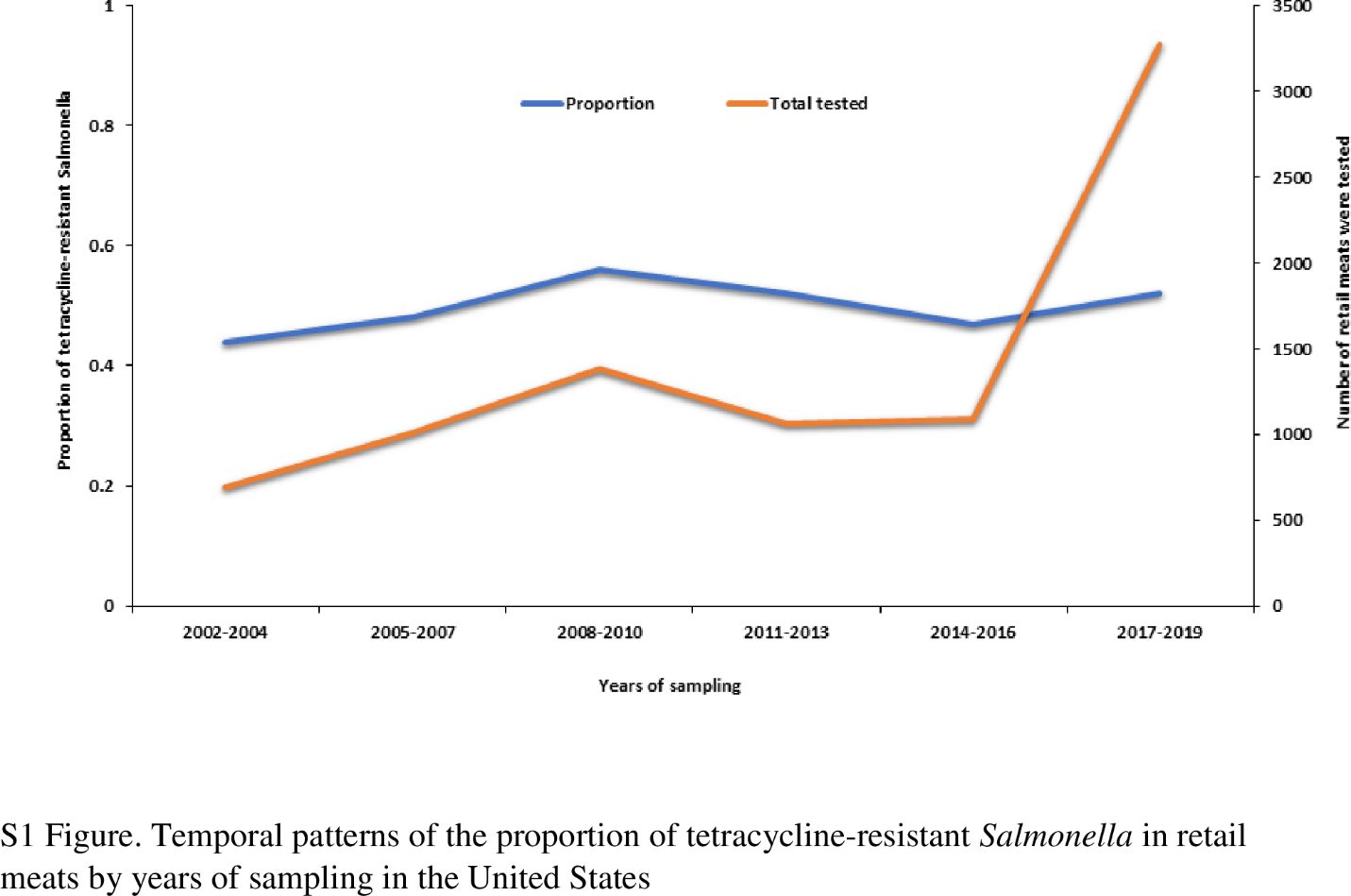


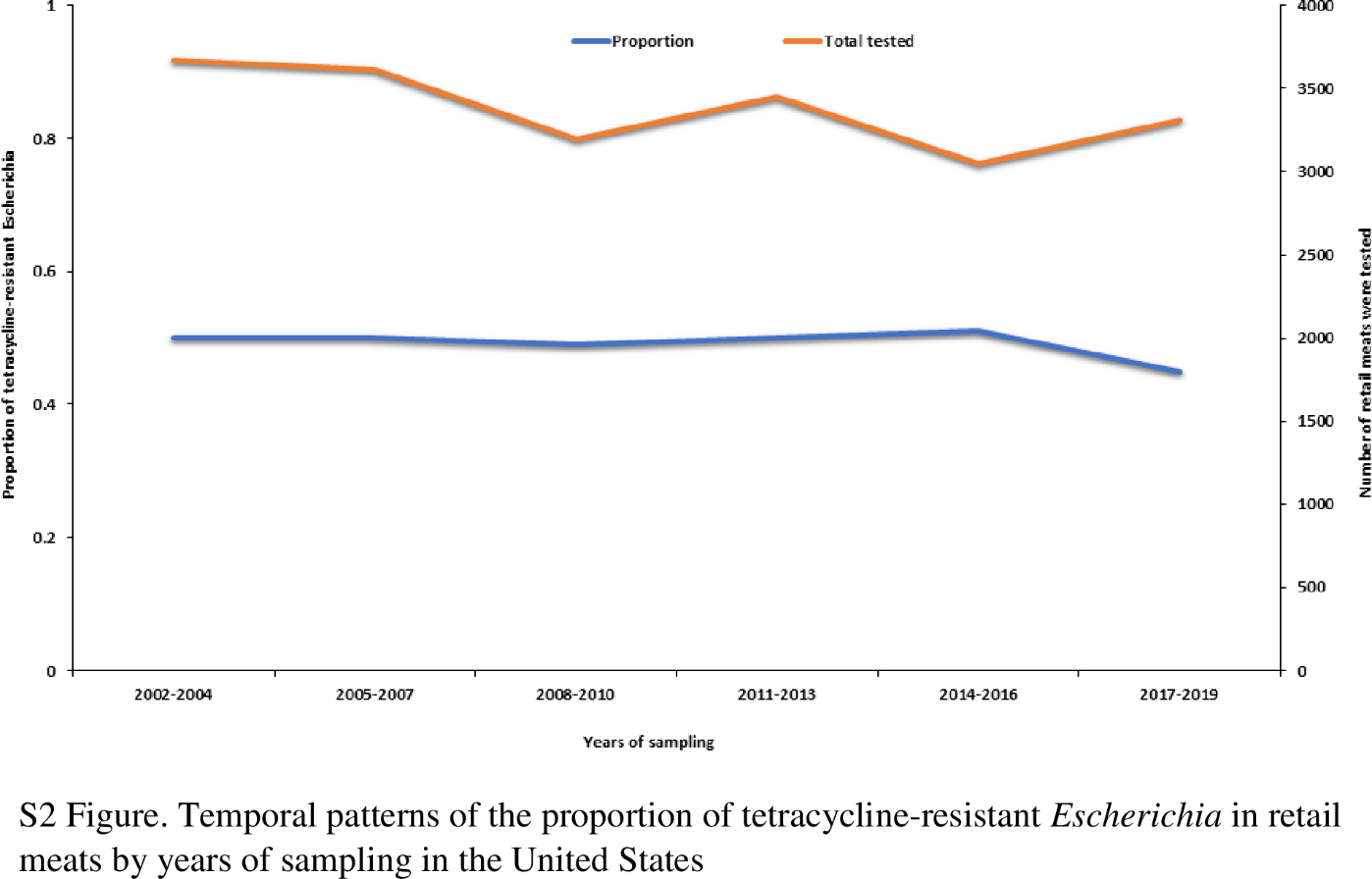


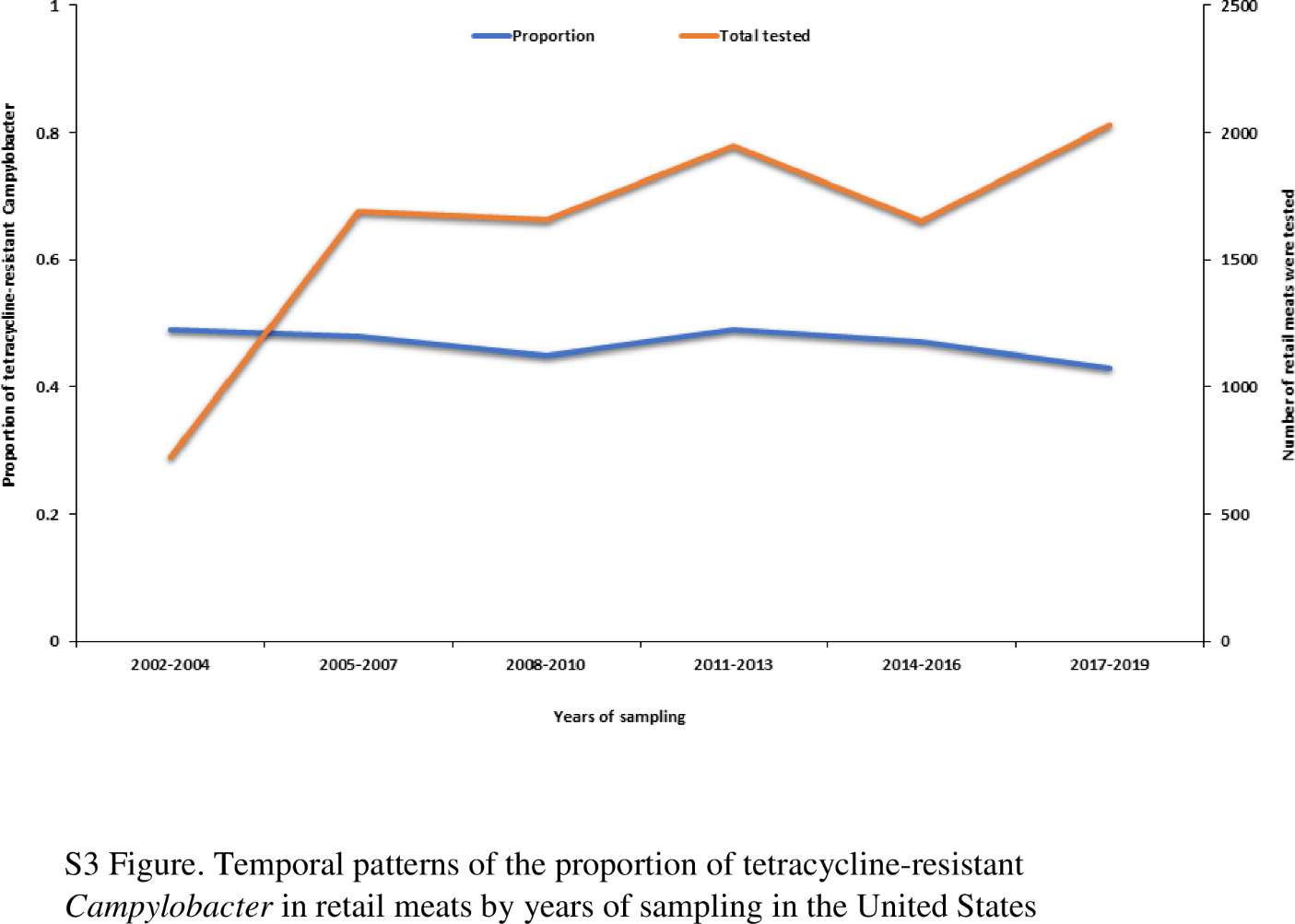


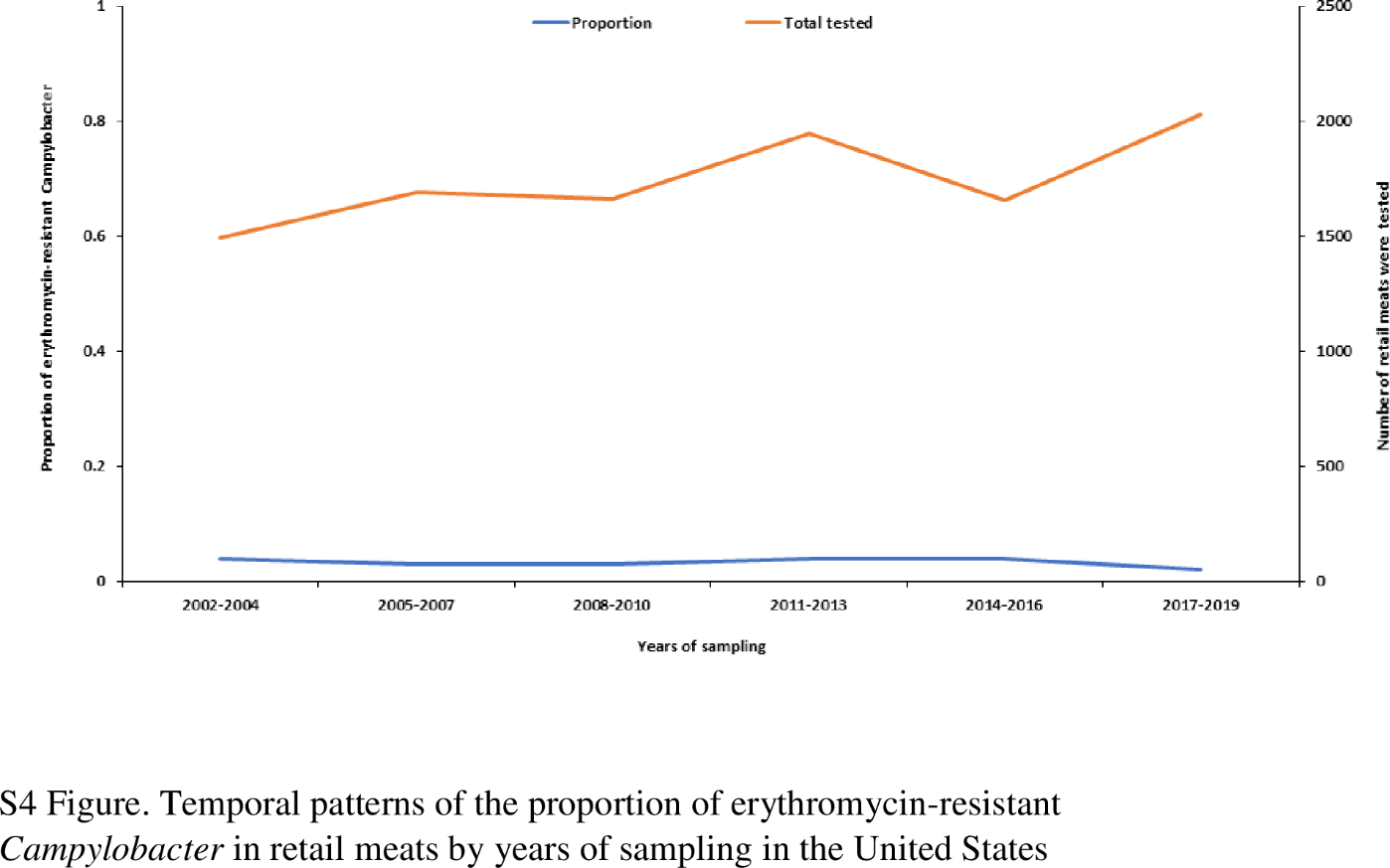

Supplement: S1 File — (DOCX) [file pone.0289208.s001.docx]
